# (*E*)-*N*-[2-(Biphenyl-4-ylvin­yl)phen­yl]furan-2-carboxamide

**DOI:** 10.1107/S1600536808034569

**Published:** 2008-10-25

**Authors:** Chin Hui Kee, Noel F. Thomas, Azhar Ariffin, Khalijah Awang, Seik Weng Ng

**Affiliations:** a Department of Chemistry, University of Malaya, 50603 Kuala Lumpur, Malaysia

## Abstract

In the title mol­ecule, C_25_H_19_NO_2_, the furyl ring is twisted by 46.3 (1)° with respect to the phenyl­ene ring bearing the amido group. In the stilbene unit, the two phenyl­ene rings (*i.e.* the rings connected through the –CH=CH– fragment) are twisted by 59.2 (1)°; in the biphenyl­ene unit, the two benzene rings are twisted by 35.5 (1)°. In the crystal structure, mol­ecules are linked by an N—H⋯O_amido_ hydrogen bond into a zigzag chain running along the *c* axis.

## Related literature

For the use of radical cations in heterocyclic synthesis, see: Thomas *et al.* (2004 ▶, 2008 ▶).
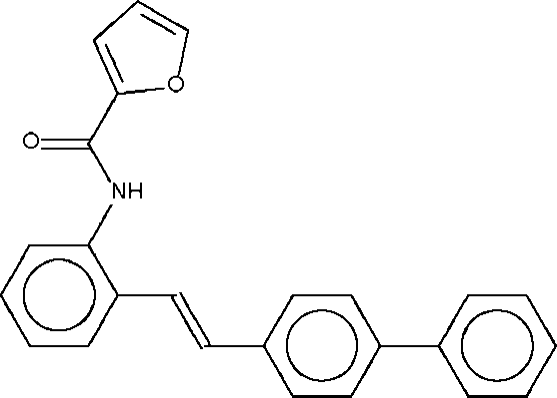


## Experimental

### 

#### Crystal data

C_25_H_19_NO_2_
*M* *_r_* = 365.41
Monoclinic, 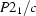

*a* = 10.9271 (2) Å
*b* = 19.7960 (4) Å
*c* = 8.7969 (1) Å
β = 92.374 (1)°
*V* = 1901.25 (6) Å^3^
*Z* = 4
Mo *K*α radiation
μ = 0.08 mm^−1^
*T* = 100 (2) K
0.40 × 0.35 × 0.15 mm

#### Data collection

Bruker SMART APEX diffractometer
Absorption correction: none
13179 measured reflections
4356 independent reflections
3681 reflections with *I* > 2σ(*I*)
*R*_int_ = 0.022

#### Refinement

*R*[*F* ^2^ > 2σ(*F* ^2^)] = 0.039
*wR*(*F* ^2^) = 0.106
*S* = 1.04
4356 reflections
253 parameters
H-atom parameters constrained
Δρ_max_ = 0.30 e Å^−3^
Δρ_min_ = −0.25 e Å^−3^

### 

Data collection: *APEX2* (Bruker, 2007 ▶); cell refinement: *SAINT* (Bruker, 2007 ▶); data reduction: *SAINT*; program(s) used to solve structure: *SHELXS97* (Sheldrick, 2008 ▶); program(s) used to refine structure: *SHELXL97* (Sheldrick, 2008 ▶); molecular graphics: *X-SEED* (Barbour, 2001 ▶); software used to prepare material for publication: *publCIF* (Westrip, 2008 ▶).

## Supplementary Material

Crystal structure: contains datablocks global, I. DOI: 10.1107/S1600536808034569/lh2714sup1.cif

Structure factors: contains datablocks I. DOI: 10.1107/S1600536808034569/lh2714Isup2.hkl

Additional supplementary materials:  crystallographic information; 3D view; checkCIF report

## Figures and Tables

**Table 1:** Hydrogen-bond geometry (Å, °)

*D*—H⋯*A*	*D*—H	H⋯*A*	*D*⋯*A*	*D*—H⋯*A*
N1—H1⋯O2^i^	0.88	2.05	2.903 (1)	163

Symmetry code: (i) 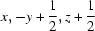.

**Fig. 1.**
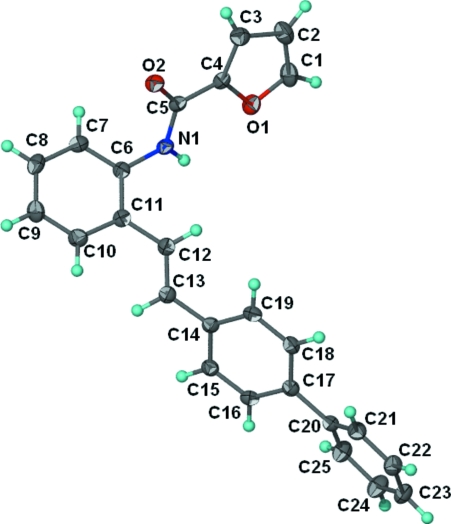
Thermal ellipsoid plot (Barbour, 2001) of C25H19NO2 at the 70% probability level. Hydrogen atoms are drawn as spheres of arbitrary radiius.

## supplementary crystallographic information

### Comment

In our earlier studies, we reported the synthesis of some stilbene carboxamides whose radical chemistry we investigated (Thomas *et al.*, 2004, 2008). In the present study, we have synthesized a new stilbene carboxamide that incorporates a furan unit (Scheme I, Fig. 1).

### Experimental

*N*-(2-Iodophenyl)furan-2-carboxamide (0.37 g, 1.2 mmol) was dissolved in DMF (20 ml) under a nitrogen atmosphere. The solution was heated to 373 K. Palladium acetate (3.2 mg, 0.014 mmol) was added followed by triethylamine (0.65 ml, 4.7 mmol) and 4-vinylbiphenyl (0.20 g, 1.21 mmol). The mixture was further heated for an hour. The solution was cooled and then mixed with saturated sodium chloride. The organic compound was extracted with ethyl acetate. The ethyl acetate solution was dried with sodium sulfate. The solvent was evaporated and the product purified by column chromatography. Single crystals were obtained by recrystallization from petroleum ether/dichloromethane.

### Refinement

Carbon- and nitrogen- bound H-atoms were placed in calculated positions (C—H 0.95, N–H 0.88 Å) and were included in the refinement in the riding model approximation, with *U*(H) set to 1.2*U*(C,*N*).

### Crystal data

**Table:**

C_25_H_19_NO_2_	*F*(000) = 768
*M**_r_* = 365.41	*D*_x_ = 1.277 Mg m^−^^3^
Monoclinic, *P*2_1_/*c*	Mo *K*α radiation, λ = 0.71073 Å
Hall symbol: -P 2ybc	Cell parameters from 5580 reflections
*a* = 10.9271 (2) Å	θ = 2.5–28.2°
*b* = 19.7960 (4) Å	µ = 0.08 mm^−^^1^
*c* = 8.7969 (1) Å	*T* = 100 K
β = 92.374 (1)°	Prism, colorless
*V* = 1901.25 (6) Å^3^	0.40 × 0.35 × 0.15 mm
*Z* = 4	

### Data collection

**Table:**

Bruker SMART APEX diffractometer	3681 reflections with *I* > 2σ(*I*)
Radiation source: fine-focus sealed tube	*R*_int_ = 0.022
graphite	θ_max_ = 27.5°, θ_min_ = 1.9°
ω scans	*h* = −14→13
13179 measured reflections	*k* = −25→25
4356 independent reflections	*l* = −11→11

### Refinement

**Table:**

Refinement on *F*^2^	Primary atom site location: structure-invariant direct methods
Least-squares matrix: full	Secondary atom site location: difference Fourier map
*R*[*F*^2^ > 2σ(*F*^2^)] = 0.039	Hydrogen site location: inferred from neighbouring sites
*wR*(*F*^2^) = 0.106	H-atom parameters constrained
*S* = 1.04	*w* = 1/[σ^2^(*F*_o_^2^) + (0.0529*P*)^2^ + 0.6489*P*] where *P* = (*F*_o_^2^ + 2*F*_c_^2^)/3
4356 reflections	(Δ/σ)_max_ = 0.001
253 parameters	Δρ_max_ = 0.30 e Å^−^^3^
0 restraints	Δρ_min_ = −0.25 e Å^−^^3^

### Special details

**Table:**

Geometry. All e.s.d.'s (except the e.s.d. in the dihedral angle between two l.s. planes) are estimated using the full covariance matrix. The cell e.s.d.'s are taken into account individually in the estimation of e.s.d.'s in distances, angles and torsion angles; correlations between e.s.d.'s in cell parameters are only used when they are defined by crystal symmetry. An approximate (isotropic) treatment of cell e.s.d.'s is used for estimating e.s.d.'s involving l.s. planes.

### Fractional atomic coordinates and isotropic or equivalent isotropic displacement parameters (Å^2^)

**Table:**

	*x*	*y*	*z*	*U*_iso_*/*U*_eq_	
O1	0.69066 (8)	0.17866 (4)	0.88070 (9)	0.0218 (2)	
O2	0.56344 (8)	0.18549 (4)	0.50228 (9)	0.01969 (19)	
N1	0.52573 (9)	0.25519 (5)	0.70226 (11)	0.0173 (2)	
H1	0.5441	0.2651	0.7981	0.021*	
C1	0.76833 (12)	0.13051 (7)	0.93850 (15)	0.0263 (3)	
H1A	0.8056	0.1316	1.0380	0.032*	
C2	0.78542 (12)	0.08127 (7)	0.83672 (16)	0.0274 (3)	
H2	0.8347	0.0421	0.8510	0.033*	
C3	0.71470 (11)	0.09950 (6)	0.70252 (15)	0.0221 (3)	
H3	0.7080	0.0751	0.6096	0.027*	
C4	0.65936 (11)	0.15860 (6)	0.73503 (13)	0.0171 (2)	
C5	0.57830 (10)	0.20106 (6)	0.63762 (13)	0.0164 (2)	
C6	0.44068 (11)	0.29656 (6)	0.61664 (13)	0.0168 (2)	
C7	0.34268 (11)	0.26554 (6)	0.53871 (14)	0.0205 (3)	
H7	0.3306	0.2183	0.5488	0.025*	
C8	0.26273 (11)	0.30299 (7)	0.44657 (14)	0.0228 (3)	
H8	0.1971	0.2814	0.3914	0.027*	
C9	0.27890 (12)	0.37245 (7)	0.43514 (14)	0.0237 (3)	
H9	0.2252	0.3984	0.3707	0.028*	
C10	0.37337 (11)	0.40363 (6)	0.51779 (14)	0.0213 (3)	
H10	0.3821	0.4513	0.5114	0.026*	
C11	0.45663 (11)	0.36676 (6)	0.61073 (13)	0.0172 (2)	
C12	0.55685 (11)	0.40039 (6)	0.69725 (13)	0.0176 (2)	
H12	0.6298	0.3755	0.7193	0.021*	
C13	0.55134 (11)	0.46414 (6)	0.74683 (13)	0.0182 (2)	
H13	0.4776	0.4882	0.7240	0.022*	
C14	0.64845 (11)	0.50049 (6)	0.83315 (13)	0.0168 (2)	
C15	0.61548 (11)	0.55136 (6)	0.93372 (13)	0.0178 (2)	
H15	0.5313	0.5613	0.9446	0.021*	
C16	0.70291 (11)	0.58746 (6)	1.01760 (13)	0.0179 (2)	
H16	0.6778	0.6198	1.0896	0.021*	
C17	0.82804 (11)	0.57700 (6)	0.99805 (13)	0.0173 (2)	
C18	0.86116 (11)	0.52600 (6)	0.89750 (13)	0.0185 (2)	
H18	0.9454	0.5175	0.8830	0.022*	
C19	0.77344 (11)	0.48767 (6)	0.81860 (13)	0.0184 (2)	
H19	0.7984	0.4523	0.7539	0.022*	
C20	0.91995 (11)	0.62106 (6)	1.07784 (13)	0.0177 (2)	
C21	1.02722 (11)	0.64044 (6)	1.00799 (14)	0.0204 (3)	
H21	1.0433	0.6235	0.9098	0.025*	
C22	1.11036 (11)	0.68399 (6)	1.07977 (14)	0.0224 (3)	
H22	1.1832	0.6962	1.0312	0.027*	
C23	1.08758 (12)	0.70986 (7)	1.22235 (15)	0.0237 (3)	
H23	1.1436	0.7405	1.2706	0.028*	
C24	0.98214 (13)	0.69059 (7)	1.29408 (15)	0.0274 (3)	
H24	0.9665	0.7078	1.3922	0.033*	
C25	0.89957 (11)	0.64639 (7)	1.22295 (14)	0.0234 (3)	
H25	0.8282	0.6332	1.2735	0.028*	

### Atomic displacement parameters (Å^2^)

**Table:**

	*U*^11^	*U*^22^	*U*^33^	*U*^12^	*U*^13^	*U*^23^
O1	0.0244 (4)	0.0254 (5)	0.0155 (4)	−0.0007 (4)	−0.0020 (3)	0.0003 (3)
O2	0.0252 (4)	0.0194 (4)	0.0144 (4)	0.0002 (3)	−0.0003 (3)	−0.0011 (3)
N1	0.0219 (5)	0.0164 (5)	0.0134 (4)	−0.0005 (4)	−0.0018 (4)	−0.0020 (4)
C1	0.0239 (6)	0.0339 (7)	0.0208 (6)	0.0012 (5)	−0.0029 (5)	0.0074 (5)
C2	0.0232 (6)	0.0279 (7)	0.0311 (7)	0.0034 (5)	0.0011 (5)	0.0086 (6)
C3	0.0225 (6)	0.0205 (6)	0.0236 (6)	−0.0005 (5)	0.0026 (5)	−0.0004 (5)
C4	0.0184 (5)	0.0181 (6)	0.0148 (5)	−0.0039 (4)	0.0013 (4)	0.0001 (4)
C5	0.0177 (5)	0.0147 (5)	0.0168 (5)	−0.0036 (4)	0.0020 (4)	0.0009 (4)
C6	0.0195 (5)	0.0177 (6)	0.0133 (5)	0.0009 (4)	0.0010 (4)	−0.0010 (4)
C7	0.0231 (6)	0.0183 (6)	0.0202 (6)	−0.0015 (5)	0.0011 (5)	−0.0032 (5)
C8	0.0209 (6)	0.0283 (7)	0.0189 (6)	−0.0029 (5)	−0.0021 (5)	−0.0039 (5)
C9	0.0242 (6)	0.0278 (7)	0.0188 (6)	0.0017 (5)	−0.0037 (5)	0.0041 (5)
C10	0.0252 (6)	0.0188 (6)	0.0198 (6)	−0.0007 (5)	0.0005 (5)	0.0030 (5)
C11	0.0199 (5)	0.0188 (6)	0.0131 (5)	−0.0010 (4)	0.0019 (4)	0.0003 (4)
C12	0.0188 (5)	0.0180 (6)	0.0161 (5)	−0.0009 (4)	0.0008 (4)	0.0025 (4)
C13	0.0188 (5)	0.0178 (6)	0.0177 (6)	−0.0010 (4)	0.0002 (4)	0.0016 (4)
C14	0.0202 (6)	0.0132 (5)	0.0170 (5)	−0.0014 (4)	−0.0006 (4)	0.0035 (4)
C15	0.0175 (5)	0.0156 (5)	0.0205 (6)	0.0002 (4)	0.0015 (4)	0.0028 (4)
C16	0.0215 (6)	0.0131 (5)	0.0191 (6)	0.0003 (4)	0.0023 (4)	0.0006 (4)
C17	0.0200 (6)	0.0149 (5)	0.0167 (5)	−0.0002 (4)	−0.0007 (4)	0.0035 (4)
C18	0.0176 (5)	0.0181 (6)	0.0198 (6)	0.0022 (4)	0.0001 (4)	0.0023 (5)
C19	0.0228 (6)	0.0147 (5)	0.0179 (6)	0.0020 (4)	0.0014 (4)	0.0002 (4)
C20	0.0188 (5)	0.0147 (5)	0.0195 (6)	0.0013 (4)	−0.0020 (4)	0.0013 (4)
C21	0.0213 (6)	0.0205 (6)	0.0195 (6)	0.0009 (5)	0.0012 (5)	−0.0007 (5)
C22	0.0194 (6)	0.0239 (6)	0.0240 (6)	−0.0029 (5)	0.0012 (5)	0.0031 (5)
C23	0.0235 (6)	0.0244 (6)	0.0228 (6)	−0.0053 (5)	−0.0039 (5)	−0.0015 (5)
C24	0.0279 (7)	0.0340 (7)	0.0202 (6)	−0.0058 (6)	0.0010 (5)	−0.0061 (5)
C25	0.0216 (6)	0.0280 (7)	0.0208 (6)	−0.0051 (5)	0.0024 (5)	−0.0005 (5)

### Geometric parameters (Å, °)

**Table:**

O1—C1	1.3607 (16)	C12—H12	0.9500
O1—C4	1.3714 (14)	C13—C14	1.4676 (16)
O2—C5	1.2340 (14)	C13—H13	0.9500
N1—C5	1.3523 (15)	C14—C15	1.3975 (16)
N1—C6	1.4295 (15)	C14—C19	1.4002 (16)
N1—H1	0.8800	C15—C16	1.3818 (17)
C1—C2	1.342 (2)	C15—H15	0.9500
C1—H1A	0.9500	C16—C17	1.4005 (16)
C2—C3	1.4302 (19)	C16—H16	0.9500
C2—H2	0.9500	C17—C18	1.3996 (17)
C3—C4	1.3528 (17)	C17—C20	1.4845 (16)
C3—H3	0.9500	C18—C19	1.3860 (17)
C4—C5	1.4706 (16)	C18—H18	0.9500
C6—C7	1.3903 (17)	C19—H19	0.9500
C6—C11	1.4019 (16)	C20—C25	1.3979 (17)
C7—C8	1.3825 (18)	C20—C21	1.3994 (16)
C7—H7	0.9500	C21—C22	1.3856 (18)
C8—C9	1.3904 (19)	C21—H21	0.9500
C8—H8	0.9500	C22—C23	1.3869 (18)
C9—C10	1.3832 (18)	C22—H22	0.9500
C9—H9	0.9500	C23—C24	1.3897 (18)
C10—C11	1.4026 (17)	C23—H23	0.9500
C10—H10	0.9500	C24—C25	1.3872 (18)
C11—C12	1.4672 (16)	C24—H24	0.9500
C12—C13	1.3373 (17)	C25—H25	0.9500
			
C1—O1—C4	105.89 (10)	C12—C13—C14	126.16 (11)
C5—N1—C6	120.70 (10)	C12—C13—H13	116.9
C5—N1—H1	119.6	C14—C13—H13	116.9
C6—N1—H1	119.6	C15—C14—C19	117.80 (11)
C2—C1—O1	111.19 (11)	C15—C14—C13	118.76 (10)
C2—C1—H1A	124.4	C19—C14—C13	123.43 (11)
O1—C1—H1A	124.4	C16—C15—C14	121.34 (11)
C1—C2—C3	106.39 (12)	C16—C15—H15	119.3
C1—C2—H2	126.8	C14—C15—H15	119.3
C3—C2—H2	126.8	C15—C16—C17	120.99 (11)
C4—C3—C2	105.94 (12)	C15—C16—H16	119.5
C4—C3—H3	127.0	C17—C16—H16	119.5
C2—C3—H3	127.0	C18—C17—C16	117.61 (11)
C3—C4—O1	110.59 (11)	C18—C17—C20	122.35 (10)
C3—C4—C5	129.40 (11)	C16—C17—C20	119.99 (10)
O1—C4—C5	119.99 (10)	C19—C18—C17	121.32 (11)
O2—C5—N1	124.22 (11)	C19—C18—H18	119.3
O2—C5—C4	118.13 (10)	C17—C18—H18	119.3
N1—C5—C4	117.65 (10)	C18—C19—C14	120.80 (11)
C7—C6—C11	120.92 (11)	C18—C19—H19	119.6
C7—C6—N1	118.52 (10)	C14—C19—H19	119.6
C11—C6—N1	120.55 (10)	C25—C20—C21	118.08 (11)
C8—C7—C6	120.50 (11)	C25—C20—C17	120.81 (10)
C8—C7—H7	119.8	C21—C20—C17	121.06 (10)
C6—C7—H7	119.8	C22—C21—C20	121.05 (11)
C7—C8—C9	119.57 (12)	C22—C21—H21	119.5
C7—C8—H8	120.2	C20—C21—H21	119.5
C9—C8—H8	120.2	C21—C22—C23	120.20 (11)
C10—C9—C8	119.85 (12)	C21—C22—H22	119.9
C10—C9—H9	120.1	C23—C22—H22	119.9
C8—C9—H9	120.1	C22—C23—C24	119.50 (12)
C9—C10—C11	121.74 (11)	C22—C23—H23	120.2
C9—C10—H10	119.1	C24—C23—H23	120.2
C11—C10—H10	119.1	C25—C24—C23	120.29 (12)
C6—C11—C10	117.32 (11)	C25—C24—H24	119.9
C6—C11—C12	121.45 (11)	C23—C24—H24	119.9
C10—C11—C12	121.22 (11)	C24—C25—C20	120.85 (11)
C13—C12—C11	123.68 (11)	C24—C25—H25	119.6
C13—C12—H12	118.2	C20—C25—H25	119.6
C11—C12—H12	118.2		
			
C4—O1—C1—C2	−0.80 (14)	C10—C11—C12—C13	28.50 (17)
O1—C1—C2—C3	0.80 (15)	C11—C12—C13—C14	−179.77 (10)
C1—C2—C3—C4	−0.48 (14)	C12—C13—C14—C15	−150.97 (12)
C2—C3—C4—O1	0.00 (14)	C12—C13—C14—C19	30.06 (18)
C2—C3—C4—C5	178.41 (11)	C19—C14—C15—C16	−0.73 (17)
C1—O1—C4—C3	0.47 (13)	C13—C14—C15—C16	−179.76 (10)
C1—O1—C4—C5	−178.11 (10)	C14—C15—C16—C17	3.81 (17)
C6—N1—C5—O2	4.14 (17)	C15—C16—C17—C18	−3.75 (17)
C6—N1—C5—C4	−176.54 (10)	C15—C16—C17—C20	173.84 (10)
C3—C4—C5—O2	−6.48 (18)	C16—C17—C18—C19	0.74 (17)
O1—C4—C5—O2	171.80 (10)	C20—C17—C18—C19	−176.79 (11)
C3—C4—C5—N1	174.16 (12)	C17—C18—C19—C14	2.28 (18)
O1—C4—C5—N1	−7.56 (15)	C15—C14—C19—C18	−2.28 (17)
C5—N1—C6—C7	51.61 (15)	C13—C14—C19—C18	176.70 (11)
C5—N1—C6—C11	−127.90 (12)	C18—C17—C20—C25	−148.23 (12)
C11—C6—C7—C8	3.72 (17)	C16—C17—C20—C25	34.29 (17)
N1—C6—C7—C8	−175.79 (10)	C18—C17—C20—C21	34.12 (17)
C6—C7—C8—C9	−1.66 (18)	C16—C17—C20—C21	−143.35 (12)
C7—C8—C9—C10	−1.08 (18)	C25—C20—C21—C22	−0.62 (18)
C8—C9—C10—C11	1.85 (18)	C17—C20—C21—C22	177.09 (11)
C7—C6—C11—C10	−2.91 (16)	C20—C21—C22—C23	−0.77 (19)
N1—C6—C11—C10	176.59 (10)	C21—C22—C23—C24	1.4 (2)
C7—C6—C11—C12	177.55 (10)	C22—C23—C24—C25	−0.7 (2)
N1—C6—C11—C12	−2.95 (16)	C23—C24—C25—C20	−0.7 (2)
C9—C10—C11—C6	0.14 (17)	C21—C20—C25—C24	1.35 (19)
C9—C10—C11—C12	179.68 (11)	C17—C20—C25—C24	−176.37 (12)
C6—C11—C12—C13	−151.98 (12)		

### Hydrogen-bond geometry (Å, °)

**Table:**

*D*—H···*A*	*D*—H	H···*A*	*D*···*A*	*D*—H···*A*
N1—H1···O2^i^	0.88	2.05	2.903 (1)	163

Symmetry codes: (i) *x*, −*y*+1/2, *z*+1/2.
